# Application of a new blood flow regulator in aortic endovascular therapy

**DOI:** 10.1186/s13019-020-1081-x

**Published:** 2020-02-28

**Authors:** Chang Liu, Yun-xing Xue, Dong-jin Wang, Qing Zhou

**Affiliations:** Department of Thoracic and Cardiovascular Surgery, Nanjing Drum Tower Hospital, Nanjing University Medical School, 321 Zhongshan Road, Nanjing, 210000 Jiangsu China

**Keywords:** Aorta, Stent, Endoleak

## Abstract

**Background:**

Endovascular repair involving branches of the aorta is still difficult in clinical treatment. A new type of blood flow regulator has been used in thoracic endovascular aortic repair/endovascular aortic repair in our centre, and the effects were followed and analysed.

**Methods:**

From March 2014 to January 2015, 14 patients with Stanford type B aortic dissection or penetrating ulcers and aortic arch pseudoaneurysms were consecutively enrolled. All patients were evaluated and underwent endovascular repair. The average age of these patients was 59 ± 14 years (34–76 years old, median 62 years), and there were 12 males and 2 females. The blood flow regulator was a self-expanding membrane-supported artificial blood vessel. The film was made from polyester that was formed into a mesh 1 mm^2^ in size. The metal stent used was made of nickel-titanium alloy.

**Results:**

The success rate for the technique was 100%. All patients underwent postoperative aortic CTA and had type III endoleak. There were no deaths and no instances of stroke, transient ischemic attack (TIA), hemiplegia, paraplegia or other central nervous system complications, and there were no left upper limb ischaemia symptoms in the group. The average follow-up time was 14.7 ± 3.6 months. One patient died of sudden death 4 months after the operation. One patient died due to abdominal aortic aneurysm rupture, and the other 12 patients survived. The survival rate was 86%. The blood flow regulator covered a total of 19 branch vessels (the intercostal artery was not counted), of which 18 experienced smooth blood flow. One patient continued to have a type III endoleak after the operation, and the endoleak disappeared after endovascular repair.

**Conclusions:**

This clinical case series of 14 patients with percutaneous transluminal stents indicates that the blood flow regulator is safe and feasible in TEVAR surgery, providing a promising new technology.

## Background

The operation involved in aortic branch vessels is difficult and traumatic; it is not appropriate to perform open surgery in elderly patients or high-risk patients with multiple complications. Recently, with the rapid development of endovascular repair technology, the restricted area of open surgery has been expanded, and the mortality rate of open surgery has been reduced. One of the major challenges of thoracic endovascular aortic repair (TEVAR) is that it is difficult to have an appropriate anchoring area when the lesion is located next to a large branch of blood vessels. To expand the anchoring area, various techniques have been introduced, such as the direct closure of branch vessels, debranching techniques, fenestration techniques, and chimney techniques.

Here, we designed a new type of blood flow regulator based on the concept of a multilayer bare stent. This blood flow regulator can achieve results of a proper landing zone and can preserve the normal flow of branches of the aorta. We reported our preliminary experiences.

## Methods

### Patients’ enrolment

This study was approved by the Ethics Committee of Nanjing Drum Tower Hospital. From March 2014 to January 2015, 14 patients were enrolled, with an average age of 59 ± 14 years (34–76 years, median 62 years) and with 12 males and 2 females. All patients were diagnosed by aortic CTA before surgery, and the type, location, and extent of the lesion were determined. All patients underwent routine examinations, including routine blood, coagulation, liver and kidney function, electrocardiogram, echocardiography, and head CT examinations. Coronary angiography was performed in patients with typical angina symptoms or electrocardiographic ischaemic changes.

### Device

**Fig. 1:**
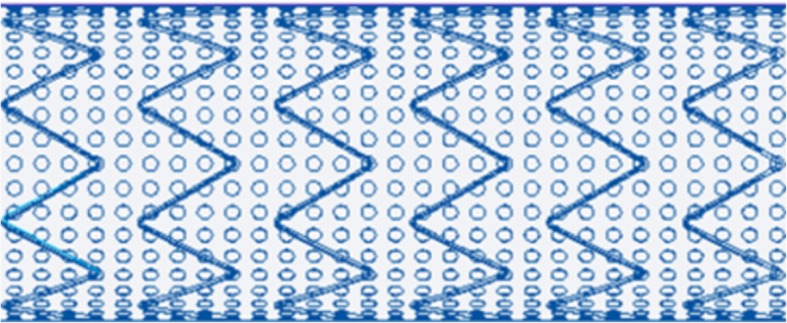
Modeled blood flow regulator

We designed a new type of blood flow regulator (Fig. 1, Yuhengjia Science and Technology Co Ltd., Beijing, China) based on the design concept of a multi-layer bare stent and the haemodynamic changes after multi-layer bare stent implantation. The blood flow regulator is a self-expanding membrane-supported artificial blood vessel, the film is made of polyester, the polyester radial and the weft are formed into a mesh 1 mm^2^ in size, and the metal stent is a nickel-titanium alloy.

### Surgical technique

The femoral artery approach was used in the operation. The type and location of the lesions were confirmed by angiography. The surgical plan was determined according to the aortic (CT angiography) CTA findings before the operation. Sixteen flow modulators (FM) were implanted during the operation. There were 10 native branched vessels (Additional file 1: Table S1). After the branch was covered, the blood flow regulator was punctured at the opening of the branch with a wire 0.018 in. in size, followed by balloon dilation (3–5 atm) at a diameter of 4 mm and then balloon dilation (3–5 atm) at a diameter of 10 mm. Angiography was then performed to observe the development of branches of blood vessels. The blood flow regulators and intraluminal-supported artificial blood vessels were located accurately, and there were no serious ischaemic events that occurred, which was considered technical success.

### Postoperative management and follow-up

All patients were given long-term oral aspirin enteric-coated tablets (100 mg qd). If there was contraindication of aspirin enteric-coated tablets, clopidogrel was taken orally for more than 12 months (75 mg qd). The patients were followed up by telephone or as an outpatient for 6 months and 1 year, and aortic CTA was performed during the follow-up. During follow-up, patients with aortic-related events were referred to the hospital at any time, and any aortic-related event was defined any event that was clearly or suspected to be associated with a treated or untreated aortic segment.

### Statistical methods

Continuous variables are expressed as medians and quartiles, and categorical variables are expressed as counts and percentages. Long-term survival rates were analysed using Kaplan-Meier curves.

## Results

### Type of aortic lesion

**Fig. 2:**
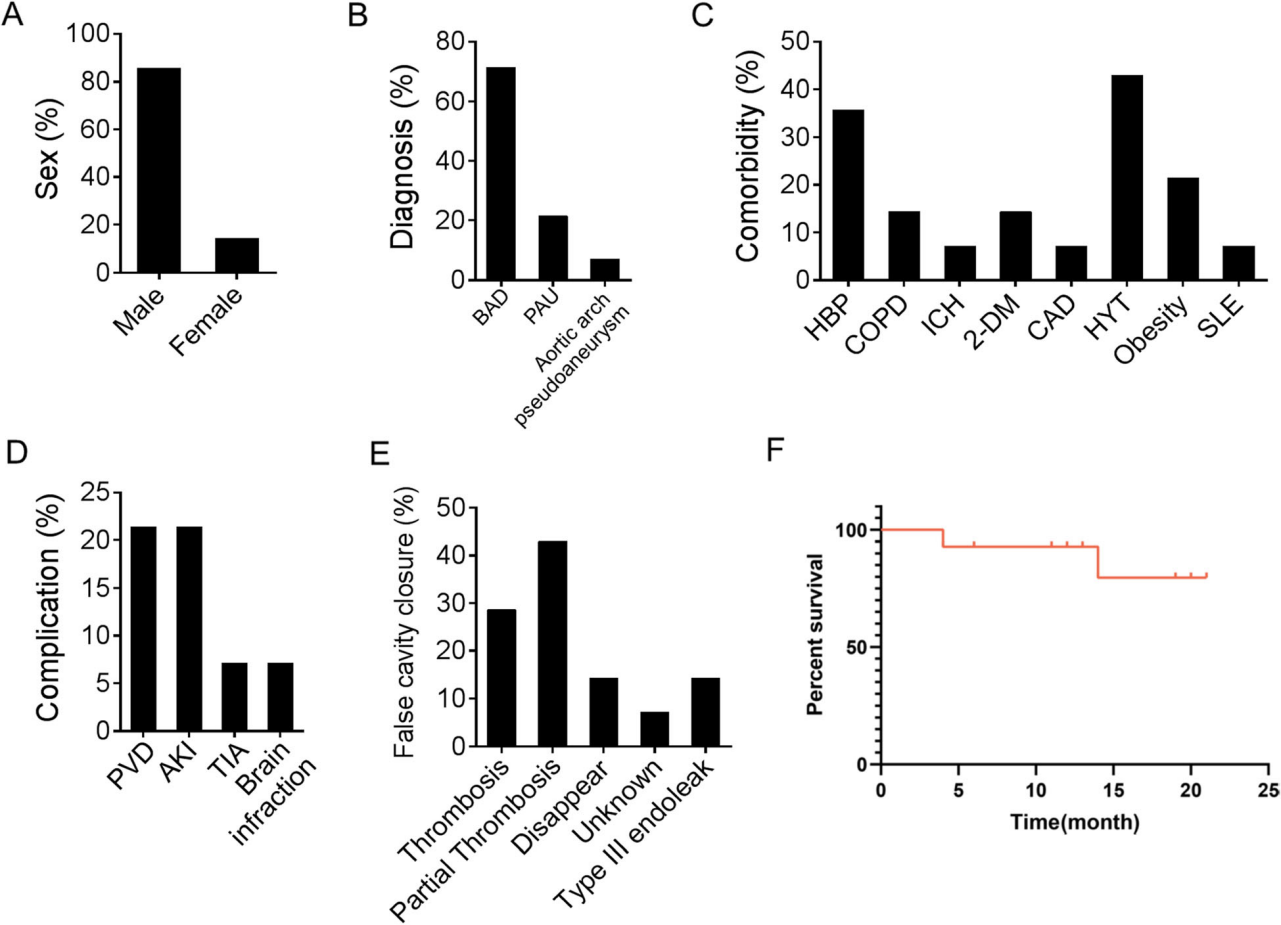
Demographics and outcome of 14 patients. **a)** Sex. **b)** Diagnose. **c)** Comorbidity. **d)** Complications. **e)** False cavity closure. **f)** Overall survival rate

**Fig. 3:**
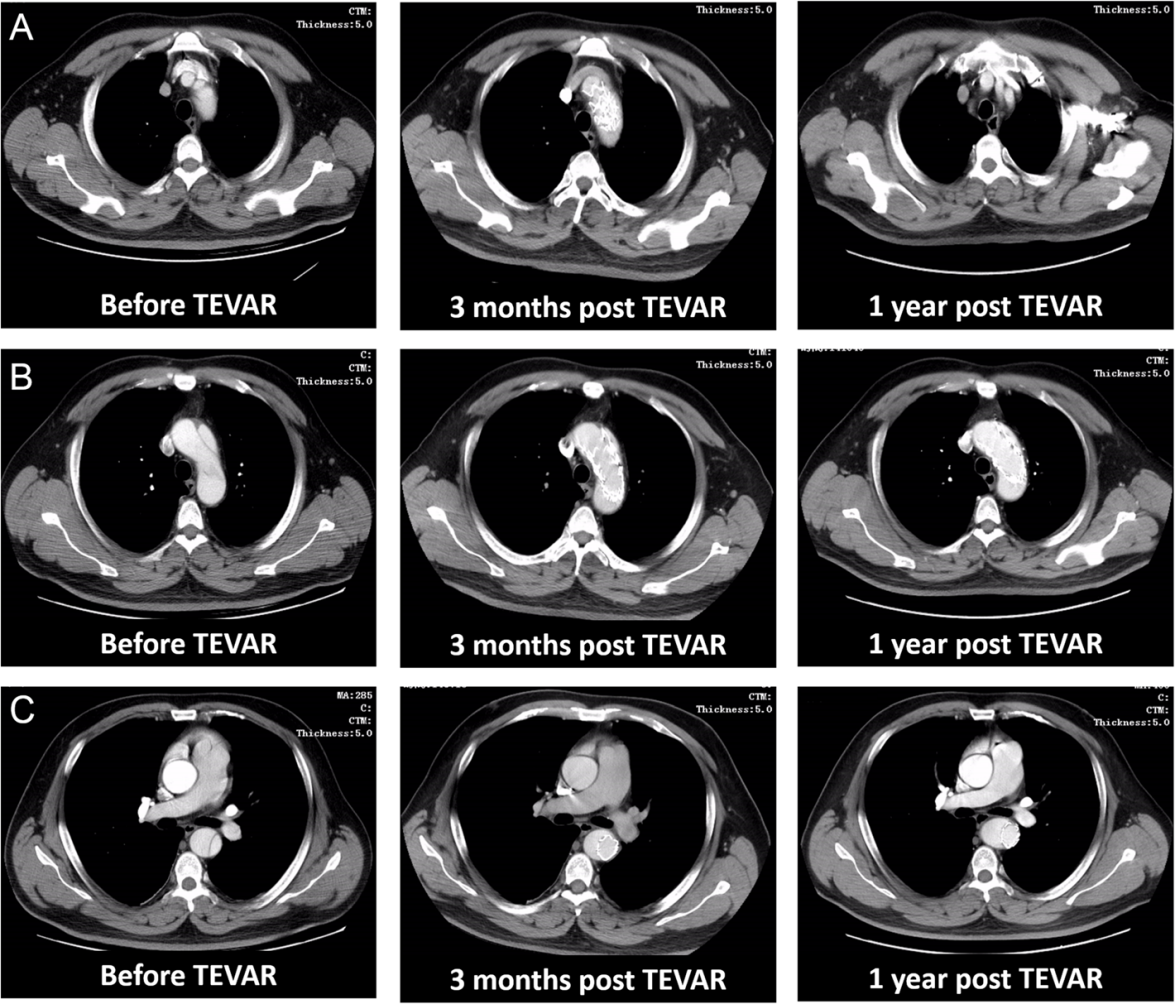
CT image of patients before and after TEVAR surgery with new blood flow regulators. **a)** Preoperative image of aortic arch branches showed the aortic dissection of a patient involving the beginning of the left subclavian artery (left panel). The centre image demonstrates the coverage of the stent graft 3 months after TEVAR, and the 12-month scan shows continuous blood flow in the aortic arch branches. **b)** The imaging of the aortic arch before surgery showed aortic dissection involving the anterior aortic arch wall (left panel). CT images 3 and 12 months after TEVAR surgery showed that aortic dissection was resected and corrected. **c)** The scan for pulmonary artery bifurcation before and after TEVAR

Of the 14 patients, 3 patients underwent acute surgery, and 11 patients underwent subacute surgery. The mean time to onset was 18 ± 6 days (6–27 days, median 19 days). There were 10 cases of Stanford type B aortic dissection (TBAD) in 14 patients: 6 primary intimal tears were located in the descending thoracic aorta and 4 in the abdominal aorta. There were 3 cases of penetrating ulcer and 2 cases of ulcer at the beginning of the descending aorta. Multiple ulcers with a distal aortic arch and descending aortic dilatation were found in 1 patient (Figs. 2 and 3, see Additional file 1: Table S1 for more details).

### In-hospital results

The technical success rate was 100%. All patients had postoperative aortic CTA with a type III endoleak. There were no deaths in the group; no central nervous system complications such as stroke, TIA, hemiplegia, and paraplegia; and no left upper limb ischaemia symptoms. Postoperative serum creatinine levels were increased in 3 patients, and postoperative liver function damage occurred in 1 patient (Additional file 1: Table S1). One case of intestinal obstruction was relieved after conservative treatment. One patient had preoperative haemorrhagic stroke with numbness of the left limbs: left upper limb and left lower limb fatigue, lameness, and no postoperative CT findings of cerebral infarction. The symptoms of the patient were associated with cerebral haemorrhage but were not related to aortic events (Additional file 1: Table S1).

### Follow-up results

All patients were followed up for an average of 14.7 ± 3.6 months (10.4–20.8 months, median 13.1 months). One patient died of unknown causes 4 months after surgery. One patient died due to abdominal aortic aneurysm rupture. The remaining 12 patients survived, and the survival rate was 91% during the 14-month follow-up. The blood flow regulator covered a total of 19 branch vessels (the intercostal artery was not counted), of which 18 demonstrated smooth blood flow. One patient had a type III endoleak after surgery and was implanted with Cook®ESBE Φ 32 × L 39 mm and a Cook®ESBE Φ 30 mm × L 58 mm (Cook, Bloomington, Ind) endovascular prostheses. The postoperative CT showed that the leak had disappeared, and the second postoperative follow-up occurred at 3 months, during which no adverse events occurred. During the follow-up period of other patients, 1 case of lower limb paralysis occurred, and muscle strength returned to normal after 1 year; 1 patient had lap gait; 2 patient had upper limb dysfunction; and 1 patient had left hand numbness that occurred 1 month after the operation and was more serious than the numbness before the operation, which might be related to preoperative cerebral haemorrhage. One case of left subclavian artery occlusion occurred 1 year after the operation, and the patient’s upper limb systolic pressure difference was 70 mmHg, the right upper limb systolic pressure was 180 mmHg, and the left upper limb systolic pressure was 110 mmHg (Additional file 1: Tables S1 and S2).

## Discussion

The open operation of aortic disease results in severe trauma and many perioperative complications; thus, open surgery is not appropriate for elderly patients or patients with multiple complications because they cannot tolerate the trauma of surgery. With the advancement of interventional techniques, TEVAR surgery has been widely used due to its advantages of being less invasive and having a rapid recovery and a low rate of mortality and morbidity. However, TEVAR surgery requires a sufficient length of normal blood vessels as the anchoring area; the presence of aortic branch vessels often results in an insufficient length of the anchoring area and limits TEVAR surgical use. In response to this problem, many solutions have been introduced, such as chimney technology, in situ fenestration technology, custom-made fenestration technology, branched stents, and multilayer bare stents [1].

Among these solutions, the most convenient one is a multilayer bare stent (known as a multilayer flow modulator, MFM). The haemodynamic changes after implantation of the MFM are aneurysms and penetrating ulcers, and turbulent flow in the aortic dissection can become laminar, making the blood flow parallel to the laminar flow of the aneurysm wall, thus eliminating turbulence, reducing pressure on the aortic wall and reducing the risk of rupture. The blood flow velocity of the aortic aneurysm or penetrating ulcer is slowed down and prone to thrombosis. The buffering effect of the thrombus further reduces the pressure on the aortic wall, and the reduction in aortic wall pressure promotes the reconstruction of the middle layer of the artery. The MFM metal stent has a mesh hole, laminar flow parallel to the branch blood vessel can be retained in the branch blood vessel, and endothelialization of the stent mesh can be inhibited. The blood flow of the branch blood vessel can be effectively preserved, where there is no branch blood vessel. It can slow the blood flow rate, facilitate the adhesion and growth of endothelial cells, and promote endothelialization of the stent mesh to isolate the tumour cavity. Haemodynamic changes after implantation of the MFM promote thrombosis in the aneurysm cavity, reduce the pressure on the aneurysm wall, and preserve branch vascular patency. TBAD can also seal the endometrial intimal opening, enlarge the true cavity, compress the false lumen and promote the thrombosis of the pseudocatheter; these mechanisms can promote the reconstruction of the aorta [2, 3].

Our new blood flow regulator produced similar haemodynamic changes in 14 patients. The technical success rate was 100%, and there were no serious liver function injuries or instances of renal failure, gastrointestinal ischaemia, or other organ perfusion symptoms in the early postoperative period. There were no acute subclavian artery ischaemia complications, such as limb pain and dizziness, and there were no central nervous system complications, such as paraplegia and apoplexy. There were 2 patients who died after 14 months of follow-up: one patient died due to abdominal aortic rupture, and the other cause was unknown (survival rate was 86% (12/14)). There were 2 cases of type-III endoleak and 1 case of aortic-related adverse events, 1 case of renal dysfunction, 1 case of repeated TIA, and 1 case of upper limb dysfunction; however, there were no complications such as stroke, paraplegia or visceral ischaemia. During the follow-up period, 90% (9/10) of patients with TBAD were treated with false lumen thromboembolism. The short-term curative effect was satisfactory, and there were no complications of covered branch embolization.

The new blood flow regulator is a commercialized product that can be used in emergency surgery without customization. It can address branch vessels via balloon dilation without implanting stent(s) into branch vessels, which avoids the bracket damage caused by the friction between pluralities of brackets in chimney technology. The blood flow regulator does not block the blood flow of the branch vessels, avoiding the ischaemic time before the fenestration of the branch vessels in the in situ fenestration technique and effectively reducing ischaemia-related complications. The blood flow regulator has the advantages of simple implantation, a low accuracy of localization, low technical difficulty, a small learning curve, a short operation time, a small dose of radiation to patients and physicians, and a small dosage of contrast agent.

The new blood flow regulator is a stent graft with a small mesh and demonstrates changes similar to the haemodynamic changes observed in aortic aneurysms with multilayer bare stents. Compared with multilayer bare stents, the new blood flow regulator has many advantages in terms of practical applications. (1) Multilayer bare stents promote thrombosis by slowing down the blood flow in the lumen of the isolated aneurysm. The thrombus of the aneurysm into the branch vessel can cause branch vessel embolization; the new type of blood flow regulator can dilate the reticular capping of branch vessels and avoid the risk of endovascular thromboembolism in isolated aneurysms after the implantation of the stent in the branch vessels. (2) The multilayer bare metal stent is not suitable for implantation in the area with thrombus because it may cause the thrombus to break off, whereas the new blood flow regulator is a mesh-shaped stent that does not cut the thrombus and isolates the thrombus from the lumen of the vessel to avoid thrombus loss. (3) The length of the multilayer bare stent is related to the diameter. Furthermore, the difficulty of accurately locating the distal end of the stent may lead to the difficulty of incomplete release, while the length of the new type of blood flow regulator is fixed after release, and the location of the distal end is accurate [4].

Blood flow through the stent will produce shear force on the stent, which requires balanced friction between the stent and the vascular wall to ensure that the stent does not shift. The friction between the stent and the vascular wall is proportional to the radial support of the stent, and the pressure generated by the radial support of the stent is inversely proportional to the length of the tumour neck. Therefore, a shorter neck must be subjected to high pressure to ensure that it is not displaced from the covered stent, and a higher pressure on the neck may lead to long-term neck dilation or even to iatrogenic aortic rupture or reverse avulsion aortic dissection. The new blood flow regulator reduces the pressure on the anchoring area by extending the proximal anchoring area, avoiding further dilation of the neck of the tumour and reducing iatrogenic aortic injury [5].

There was no paraplegia in this group of patients and no paraplegia or other spinal cord ischaemia after covering the intercostal artery, as permanent paraplegia after TEVAR is associated with the length of the thoracic aorta covered. Feezor et al. [6] found that spinal cord ischaemia after TEVAR occurred when the distance between the stent graft and the proximal end of the abdominal cavity was 17.3 ± 21.8 mm, whereas no ischaemia was observed when the distance was 63.1 ± 62.9 mm (*p* = 0.0006). The coverage length threshold was 205 mm, and the sensitivity and specificity of predicting spinal cord ischaemia after TEVAR were 80 and 92.5%, respectively. The effect of the new blood flow regulator on preventing spinal cord ischaemia was significantly better than that of the stent graft. This was a chronic occlusion of the spinal cord artery after the blood flow regulator covered the intercostal artery, and the chronic occlusion provided sufficient time for the establishment of the spinal artery collateral circulation [7]. Some studies have also suggested that planned, temporary endoleak after TEVAR can maintain spinal cord blood supply to reduce the incidence of paraplegia [8].

The new blood flow regulator can also be used in combination with stent grafts in some special cases. (1) When the length of the anchoring area is insufficient, the blood flow regulator is first placed into the normal vessel wall, the anchoring area is expanded, and then the stent graft is implanted in the flow regulator. (2) When the large tumour cavity or endometrial rupture is adjacent to the branch vessel, the blood flow regulator can be implanted first, and then the stent graft is implanted in the vicinity of the branch vessel opening, which can partially close the tumour cavity or break and can reduce the permeation stent. The amount of internal leakage promotes thrombosis in the tumour cavity or pseudocavity and promotes vascular remodelling. There were 5 patients in this group. First, a blood flow regulator was placed in the aortic arch, and then a stent graft was placed in the blood flow regulator. During the follow-up, the tumour cavity was thrombotic, and the branch vessels were patent. The combination of blood flow regulators and stent grafts can expand the surgical indications of TEVAR and achieve good results.

However, there are some defects of this new blood flow regulator, such as type III endoleak and thromboembolism. The occurrence of endoleak is mainly related to the mesh-like lamella, the size of the aortic aneurysm and the size of the aortic dissection. After the operation, the type III endoleak can be directly closed by implanting sent grafts. Otherwise, another blood flow regulator can be superimposed to reduce the mesh size. To reduce type III endoleaks, permeation stents with different mesh sizes can be applied according to the size of different tumour cavities. The mesh-like mulch covering the opening of the branch vessel may result in thrombosis in the early stage. To solve this problem, aspirin was used orally (100 mg qd) to prevent thrombosis. After the transmembrane stent was covered with endothelial cells, the probability of thrombosis was significantly reduced. Antiplatelet therapy is thought to affect the thrombosis of the tumour cavity or pseudocavity and aortic remodelling, but it can also increase the patency of branch collateral vessels. Our results showed that thromboembolism occurred when the blood flow regulator was applied to the arcuate vessels. Therefore, anticoagulation is necessary after the blood flow regulator is implanted.

The limitation of this study is the small number of patients and the short follow-up time. The effect of the regulator is good according to recent results, but further studies are needed to assess the long-term effect. Major long-term observations include the time of thrombosis of the pseudocavity or tumour cavity, the reconstruction of the aorta, the patency of branch vessels, and changes in the neck pressure. Most patients in recent studies were treated in the subacute phase. At this time, the inflammation of the aorta is lower than that in the acute phase, and the toughness of the endometrium is increased but not rigid. After TEVAR, the true cavity is fully enlarged, and the pseudocavity is compressed. The middle layer is closely adhered to the outer membrane to achieve a better therapeutic effect. The treatment of chronic interlayers remains to be addressed.

## Conclusion

In the current study, we demonstrate that the blood flow regulator, when used to treat TBAD, works by achieving results of a proper landing zone and preserving the normal flow of branches of the aorta. The blood flow regulator is a safe and feasible choice for TEVAR surgery in China. Its application will provide a promising new technology currently used for TEVAR surgery in this region.

## Supplementary information

**Additional file 1: Table S1.** Summary of case data and outcome. **Table S2.** Case data.

## Data Availability

The datasets used are available from the corresponding author on reasonable request.

## Abbreviations

2-DM: Type 2 diabetes
AKI: Acute renal failure
BAD: Stanford B aortic dissection
CAD: Coronary artery disease
COPD: Chronic obstructive pulmonary disease
CT: Computed tomography
CTA: CT angiography
FM: Flow modulators
HYT: Hypertension
ICH: Intracranial haemorrhage
MFM: Multilayer flow modulator
PAU: Penetrating ulcer
PVD: Peripheral artery disease
SLE: Systemic lupus erythematosus
TBAD: Type B aortic dissection
TEVAR: Thoracic endovascular aortic repair
TIA: Transient ischemic attack
