# Supplementary figures and images for: Application of a new blood flow regulator in aortic endovascular therapy
Source: J Cardiothorac Surg. 2020 Feb 28;15:47. doi: 10.1186/s13019-020-1081-x (PMC7048071; doi:10.1186/s13019-020-1081-x)

**Table S1. Summary of case data and outcome**


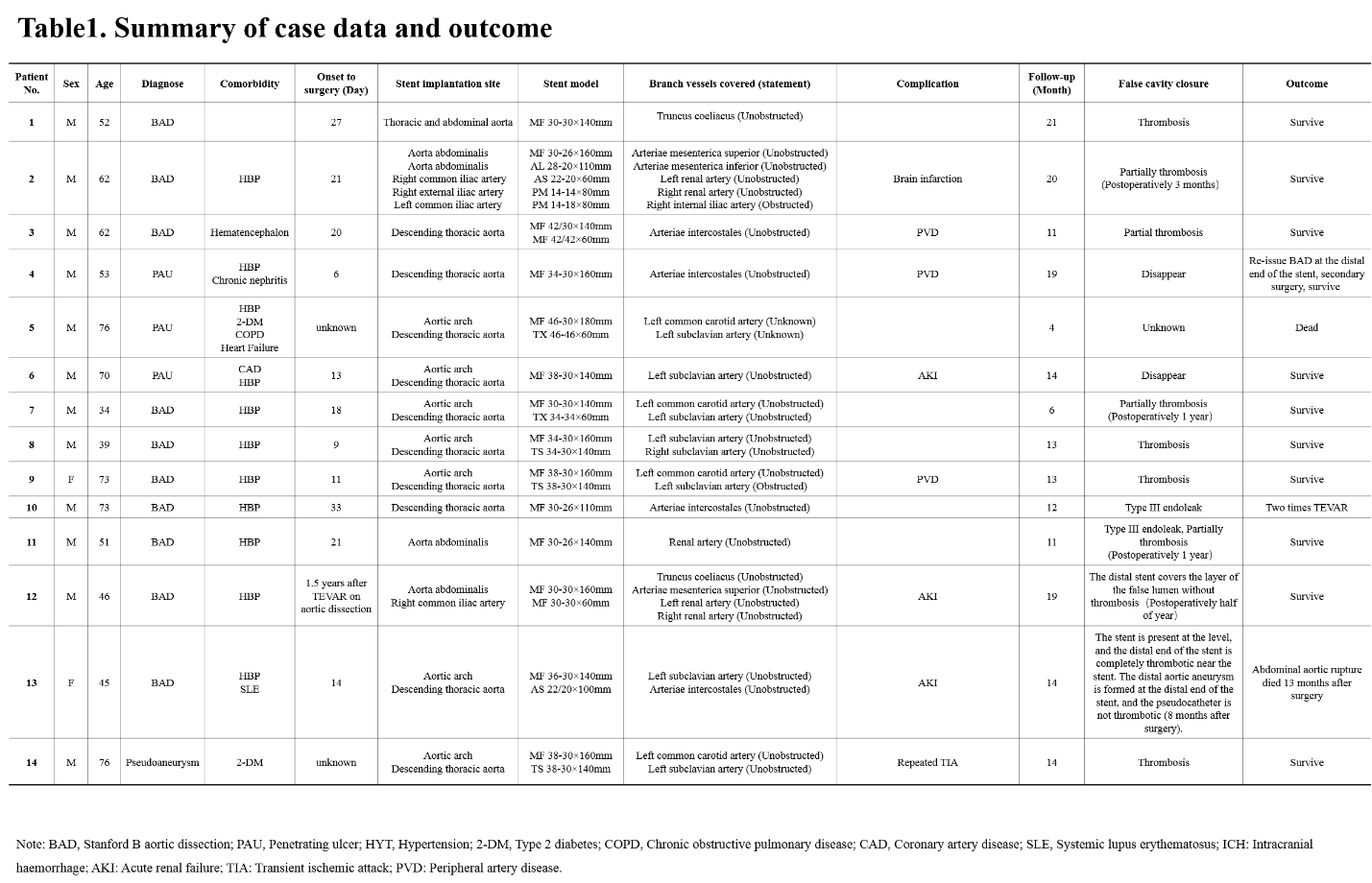


**Table S2. Case data (Continued)**


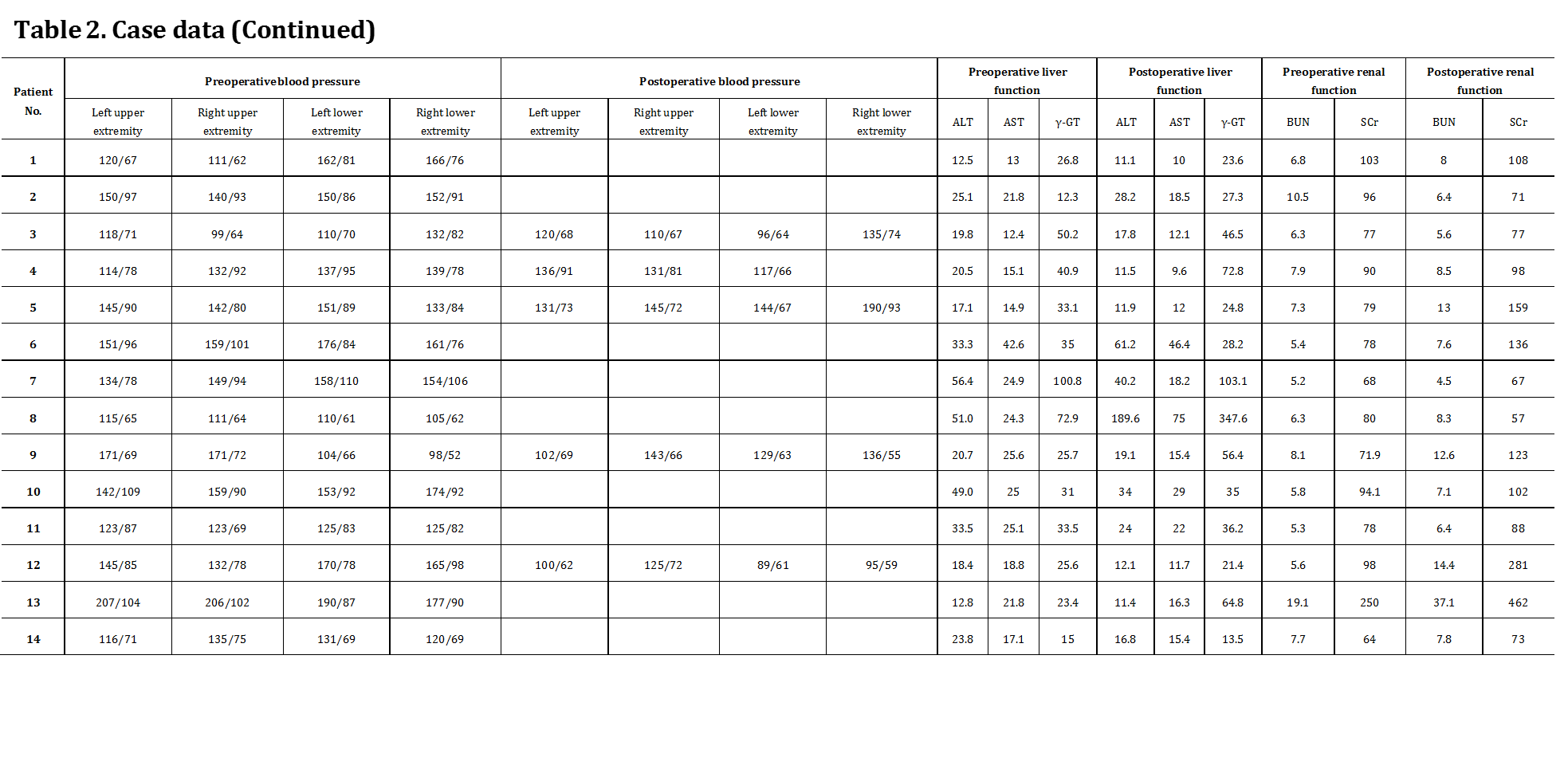

Supplement: Supplementary file 1 — Additional file 1: Table S1. Summary of case data and outcome. Table S2. Case data. [file 13019_2020_1081_MOESM1_ESM.docx]
